# A technique for pediatric total skin electron irradiation

**DOI:** 10.1186/1748-717X-7-40

**Published:** 2012-03-20

**Authors:** Qinan Bao, Brian A Hrycushko, Joseph P Dugas, Frederick H Hager, Timothy D Solberg

**Affiliations:** 1 Department of Radiation Oncology, University of Texas Southwestern Medical Center, 5801 Forest Park Rd, Dallas, TX 75390, USA; 2 Mary Bird Perkins Cancer Center, 4950 Essen Lane, Baton Rouge, LA 70809, USA; 3 Department of Physics and Astronomy, Louisiana State University, 202 Nicholson Hall, Baton Rouge, LA 70803, USA

**Keywords:** Pediatric total skin electron irradiation, Commissioning, Quality assurance, Leukemia cutis

## Abstract

**Background:**

Total skin electron irradiation (TSEI) is a special radiotherapy technique which has generally been used for treating adult patients with mycosis fungoides. Recently, two infants presented with leukemia cutis isolated to the skin requiring TSEI. This work discusses the commissioning and quality assurance (QA) methods for implementing a modified Stanford technique using a rotating harness system to position sedated pediatric patients treated with electrons to the total skin.

**Methods and Results:**

Commissioning of pediatric TSEI consisted of absolute calibration, measurement of dosimetric parameters, and subsequent verification in a pediatric patient sized cylindrical phantom using radiographic film and optically stimulated luminance (OSL) dosimeters. The depth of dose penetration under TSEI treatment condition was evaluated using radiographic film sandwiched in the phantom and demonstrated a 2 cm penetration depth with the maximum dose located at the phantom surface. Dosimetry measurements on the cylindrical phantom and in-vivo measurements from the patients suggested that, the factor relating the skin and calibration point doses (i.e., the *B*-factor) was larger for the pediatric TSEI treatments as compared to adult TSEI treatments. Custom made equipment, including a rotating plate and harness, was fabricated and added to a standard total body irradiation stand and tested to facilitate patient setup under sedated condition. A pediatric TSEI QA program, consisting of daily output, energy, flatness, and symmetry measurements as well as in-vivo dosimetry verification for the first cycle was developed. With a long interval between pediatric TSEI cases, absolute dosimetry was also repeated as part of the QA program. In-vivo dosimetry for the first two infants showed that a dose of ± 10% of the prescription dose can be achieved over the entire patient body.

**Conclusion:**

Though pediatric leukemia cutis and the subsequent need for TSEI are rare, the ability to commission the technique on a modified TBI stand is appealing for clinical implementation and has been successfully used for the treatment of two pediatric patients at our institution.

## Background

Total skin electron irradiation (TSEI) is a special radiotherapy technique which aims to deliver a uniform dose to the entire skin of a patient while sparing all other organs from a significant amount of radiation. TSEI has historically been used for the treatment of cutaneous T-cell lymphoma (mycosis fungoides), but has also been extended for the treatment of other cutaneous diseases such as Kaposi's sarcoma and scleromyxodema [1-3]. Due to the ability to achieve therapeutic dose levels to the skin with a rapid fall-off in dose beyond a shallow depth to avoid bone marrow toxicity, electron beams delivered to the total body in the energy range of 3-7 MeV (4-10 MeV at the accelerator beam exit window) have been shown to be successful for treating these superficial lesions. First proposed in the early 1950's, various TSEI treatment techniques have been evaluated and clinically implemented [4-10], and a detailed technical report on the subject has been published for the American Association of Physicists in Medicine by the American Institute of Physics [11].

Recently, two pediatric patients with recurrent acute myelogenous leukemia presenting with leukemia cutis (LC) were treated at the University of Texas Southwestern (UTSW) Medical Center at Dallas using TSEI. LC is an extramedullary leukemia where neoplastic leukocytes have infiltrated into the skin. Although uncommon, this skin manifestation can occur with most forms of leukemia. Patients typically present with multiple raised skin nodules and plaques. The isolated skin condition suggests treatment with superficial electron beam irradiation to the total skin and has previously been clinically described in case reports [12-15].

A particular challenge in implementing a clinical pediatric TSEI program is how to deliver a uniform dose to the total skin of a sedated infant. This report describes the dosimetric commissioning and quality assurance (QA) procedures for implementing a clinical TSEI program for the treatment of LC in pediatric patients using a modified Stanford technique [4,16,17]. The commissioning of fabricated setup equipment used to hold sedated pediatric patients in a modified total body irradiation (TBI) stand is also presented. To the best of our knowledge, this is the first report on the commissioning and clinical implementation of pediatric TSEI using this technique.

## Methods

The pediatric TSEI technique described below is based on a modified Stanford technique, which has been used for treating adult TSEI patients at our institution. In developing a pediatric TSEI program, the same beam configuration of six dual-fields was adopted. Due to the smaller size of pediatric patients and the sedation requirement for consistent positioning, however, additional equipment and dosimetric commissioning measurements were required. The following section describes the measurements used in commissioning our adult TSEI program and the further steps required for commissioning and performing QA tests for pediatric TSEI patients.

### Dosimetric commissioning and quality assurance

#### Adult TSEI field flatness and calibration

**Figure 1:**
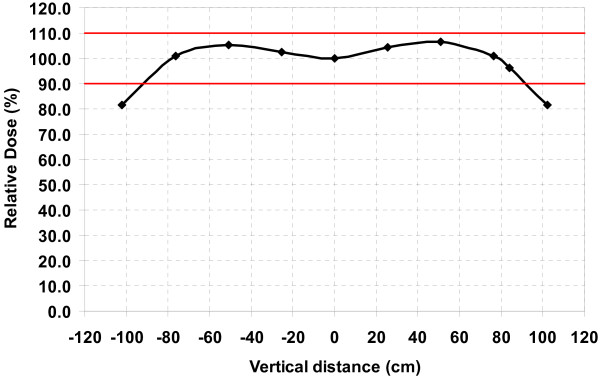
**Combined beam profile at 330 cm SSD**. The gantry was directed at ± 20° from the horizontal.

At our institution, adult TSEI patients are treated with six dual-fields (anterior, posterior, and four obliques) using gantry angles of ± 20° from the horizontal axis. These angles are used to achieve a uniform dose (± 10%) over a region of more than 170 cm while simultaneously reducing the total body dose from the mostly forward peaked contaminant bremsstrahlung photons. The combined beam profile, shown in Figure 1, was measured at the surface of solid water stacks (1 mm intrinsic ion chamber buildup) using an Exradin P11 parallel-plate ion chamber (Standard Imaging, Middleton, WI) at a distance of 330 cm SSD from a Varian Clinac 21EX linear accelerator (Varian Medical Systems, Palo Alto, CA) operating under the high dose rate TSE mode (6 MeV, 888 MU/min nominal dose rate). A 1.2 cm plexiglass scatter plate was placed 25 cm in front of the patient to provide additional electron scattering and to reduce the electron incident energy.

Absolute calibration of the machine output was performed for a single dual-field beam, using an Exradin P11 parallel-plate ion chamber under treatment conditions (330 cm SSD, 36 × 36 cm^2 ^field size, 250° and 290° gantry angles) at a depth of d_max _on the horizontal central axis according to AAPM protocol [11]. The parallel-plate chamber was first cross-calibrated against a NIST-traceable PTW 31013 cylindrical ion chamber (PTW, Hicksville, NY) in an 18 MeV electron beam, following AAPM TG-21 [18] and TG-51 [19] protocols.

The entire treatment was delivered to a Rando phantom (The Phantom Laboratory, Salem, NY). Following AAPM Report 23 [11], the multiplication factor (*B*-factor) for adult patients, which relates the treatment skin dose (*D_S_*)_*w *_to the calibration point dose (*D_P_*)_*w*_, was determined using OneDose MOSFETs (Sicel Technologies, Inc., Morrisville, NC) placed on the surface of the Rando phantom and percent depth dose data. Conceptually, this factor represents the decrease in dose for each single field due to the contribution from the two adjacent fields. The *B*-factor was used to specify the dose per field per treatment cycle and required monitor units (MU) to patients, and was also used as the starting point for MU calculations to deliver doses to a pediatric patient sized phantom described below.

### Phantom measurements for pediatric TSEI commissioning

Since the pediatric patient size is much smaller than the adult patient, it is necessary to determine whether the *B*-factor for the adult patient is applicable to the pediatric patient. A custom made cylindrical acrylic phantom of 20 cm diameter and 25 cm height was used for this evaluation. Two aluminum oxide (Al_2_O_3_) MicroStar nanoDot™ optically stimulated luminescent (OSL) dosimeters (Landauer Inc., Glenwood, IL) were placed on each of four locations (anterior, posterior, left lateral, and right lateral) at the surface of the phantom and aligned with the central axis. A surface dose of 50 cGy was delivered by manually rotating the phantom at 60° steps through each of the six TSEI positions. The required number of MUs was calculated using the *B*-factor for the adult patient. The dose received by the OSL nanoDots were read out using a MicroStar OSL reader with a luminescence-to-dose calibration curve applied.

XV film was sandwiched in the cylindrical acrylic phantom at mid-phantom height. The excess film was cut in a darkroom so that the film edges conformed to the circular surface of the phantom. 3 M Scotch black duct tape was wrapped around the phantom over the film edge to ensure the film was light-tight. The film plane was aligned with the horizontal central axis horizontal plane and a 50 cGy surface dose was delivered using the same technique as described above. The film was then developed and scanned using a Vidar VXR Dosimetry Pro film digitizer (VIDAR Systems Corporation, Herndon, VA). RIT113V5.2 software (Radiological Imaging Technology, Inc., Colorado Springs, CO) was used to apply a calibration curve to assess the depth dose distribution, dose profile, and isodose curves.

### Treatment design

#### Equipment

**Figure 2:**
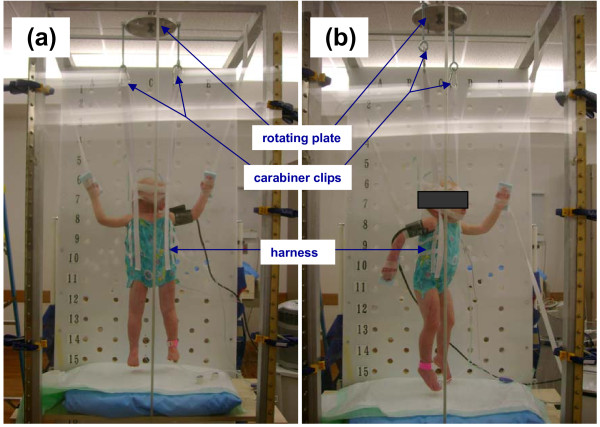
**The patient in treatment positions for (a) PA and (b) RAO locations**.

In order to position sedated pediatric patients into the six required angles required for TSEI, a rotating plate/harness system was constructed and attached to a custom made frame used for Total Body Irradiation (TBI) treatments (Figure 2). A central bar was mounted on top of the TBI frame and an aluminum rotating plate was attached to the bar. Holes were drilled on the outer rim of the rotating plate at 120 degrees increments to facilitate patient positioning at 60° interval for the 6 positions. A bolt is used to index the rotating plate to the top central bar for each different position to ensure the positioning accuracy. The entire plate can be moved along the central bar, which is parallel to the beam axis, to maintain a constant patient to spoiler distance of 25 cm at the central beam axis for each position over the course of the treatment.

A custom made harness was sewn to hold the patient vertically. The harness only covers the trunk of the patient body, leaving arms and legs uncovered. The harness attaches to the rotating plate with four belts which loop through carabiner clips. The lengths of the belts were constructed such that the patient umbilicus is centered along the horizontal beam axis. The harness was made from a single layer cloth to minimize the attenuation of the electron beam yet maintain enough strength to support an infant. To ensure patient safety during treatment, the strength of the harness was verified by placing weights inside the harness while hanging on the rotating plate for at least one hour.

### Treatment procedure

Two patients (17 months and 12 months old) have been treated since commissioning this pediatric TSEI technique. Each patient was scheduled to receive 16 Gy over the entire treatment course for 8 cycles as described in case reports [13,14]. Each cycle consists of two treatment days with three dual-fields each: AP, LPO, RPO on the first day and PA, LAO, RAO on the second day. During treatment days, morning machine output quality assurance was performed using the high dose rate TSE delivery mode using a Daily QA3 detector array (Sun Nuclear Corporation, Melbourne, FL) to verify output consistency.

Prior to each treatment, the stand was moved into a preset position and the source-to-spoiler distance was measured to ensure the correct stand position. The patient was anesthetized and placed within the harness while on a gurney. The patient was monitored for hemodynamic status, pulse oximetry, and nasal end tidal carbon dioxide levels during the entire treatment. The patient was carefully lifted into place while the harness belts were clipped onto the carabiners attached to the rotating plate. The patient's head was either supported by a piece of acrylic plate placed opposite of the beam or taped in place to the harness belts. Patient arms were positioned in similar positions as for adult TSEI patients using Velcro strips which were looped around the TBI frame and attached to a Styrofoam pad taped to the patient's hand opposite of the beam (Figure 2). After the patient AP position was set, the distance from the patient to the spoiler was measured, and the rotating plate was moved along the bars of the TBI stand to the desired 25 cm spoiler-to-patient distance. The required number of MUs was delivered and then the patient was rotated to each posterior oblique position for each subsequent delivery to complete the first half of the cycle.

As part of routine quality assurance tests, OSL dosimeters were placed on the patient's skin at multiple locations. Doses for the first two days of treatment were measured and combined to give the total dose delivered during one cycle, and this was compared against the prescription dose of 200 cGy per cycle. Based on the OSL measurements, the *B*-factor and the required MUs were adjusted if needed in order to meet our absolute dose and dose uniformity criteria.

## Results

### Phantom measurements for pediatric TSEI commissioning

Two nanoDots were placed on each of four locations on the cylindrical acrylic phantom and uniformly irradiated to a nominal dose of 50 cGy, based on the adult TSEI *B*-factor of 2.414, using the six dual-field technique. Each nanoDot was read out 3 times and the average of the 3 readings is shown in Table 1.

**Table 1:** OSL detector measured dose on 20 cm diameter cylindrical phantom following delivery of a prescribed 50 cGy

OSL Detector Location	NanoDot 1 Dose (cGy)	NanoDot 2 Dose (cGy)	Average Dose (cGy)	% Diff from Prescription
AP	54.3	53.3	53.8	7.6

Lt Lat	53.5	54.4	54.0	8.0

PA	53.9	53.1	53.5	7.0

Rt Lat	54.5	52.4	53.4	6.8

**Average**			**53.7**	**7.3**

The dose delivered to the surface of the 20 cm diameter phantom was measured to be 53.7 cGy on average at the four locations around the phantom periphery, 7.3% higher than the nominally delivered dose. Given the intrinsic 5% accuracy of the OSL dosimeters, the dose uniformity requirement, and the fact that these measurements were not patient specific, the *B*-factor was not adjusted for subsequent patient MU calculations.

**Figure 3:**
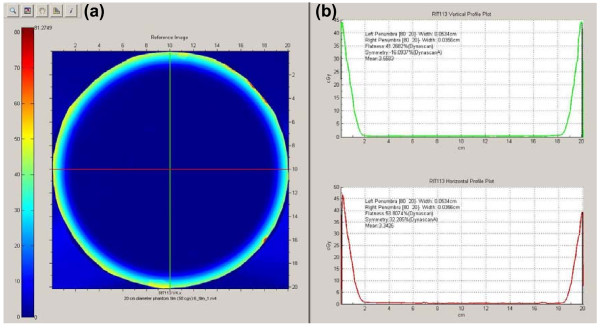
**Film measurement for the composite TSEI beam**. (**a**) An XV film was uniformly irradiated with 50 cGy dose and (**b**) two orthogonal profiles indicates the dose penetration of the TSEI beam.

While the OSL dosimeters were used for absolute dose measurements, film was used for relative dose measurements because a batch specific calibration curve and carefully controlled development conditions were not used. Figure 3a shows the dose distribution on a central axial slice of the 20 cm diameter cylindrical phantom measured by KODAK X-OMAT V (XV) film following the delivery of the MUs calculated to deliver 50 cGy. Two orthogonal lines were drawn using RIT software and the profiles are shown in Figure 3b. Based on the dose profile of the film, it can be seen that the periphery of the film was uniformly irradiated from the degraded 6 MeV electron beams. The penetration of electrons after the spoiler has a maximum range of approximately 2 cm, with the maximum dose occurring at the surface of the phantom. The cold surface dose artifact on the right-hand side of the horizontal profile is believed to be caused by misalignment of the film edge with the cylindrical phantom edge. Perfect alignment was difficult to obtain when cutting and matching the film with the phantom in a dark-room.

### Patient in-vivo dosimetry

During the first two treatment days (first cycle) of the first pediatric TSEI patient, patient specific in-vivo dosimetry using OSL detectors was performed for different locations on the body surface to verify dose calculations and the *B*-factor. The resulting dose measurements are summarized in Table 2. The average dose was 222.2 cGy over all measured locations, 11.1% higher on average than the prescription dose. This value was consistent with the 7.3% higher dose measured on the cylindrical phantom, thus the value of the *B*-factor was increased by 10% (from 2.414 to 2.655) and the MUs prescribed for each beam were correspondingly decreased by 10%. Due to the smaller diameter of the 20 cm cylindrical phantom and pediatric patients, it is reasonable that the *B*-factor increases compared to the adult TSEI technique. For the six dual-field beam arrangement, the dose contribution at any one location is primarily from three of the six dual-fields. For example, the dose contribution to the umbilicus point is primarily from the AP, LAO and RAO beams. The AP beam will contribute the same amount of dose to the umbilicus point regardless of the patient size, but for a smaller diameter phantom or patient at the same SSD, the two oblique beams will contribute more dose to the umbilicus point, resulting in a higher *B*-factor. This inverse relationship of *B*-factor with patient diameter has been reported previously [20].

**Table 2:** OSL detector dose measurements for cycle one of first patient

OSL Detector Location	AP + LPO + RPO Day 1 Dose (cGy)	PA + LAO + RAO Day 2 Dose (cGy)	Total Dose (cGy)	% Diff from Prescription
Forehead	106.2	119.1	225.3	12.7

Posterior of head	152.1	96.2	248.3	24.2

Sternum	78.9	136.9	215.8	7.9

Posterior of sternum	137.8	85.9	223.7	11.9

Umbilicus	80.5	132.7	213.2	6.6

Posterior of Umbilicus	146.9	78.7	225.6	12.8

Left thigh	75.8	139.1	214.9	7.5

Posterior Left Thigh	145.9	81.5	227.4	13.7

Right thigh	77.1	133.7	210.8	5.4

Posterior Right Thigh	130.0	86.5	216.5	8.3

Right Foot	84.6	141.6	226.2	13.1

Bottom of Right Foot	135.9	82.6	218.5	9.3

**Average**			**222.2**	**11.1**

Following adjustment of the MUs, in-vivo dosimetry using OSL detectors was repeated as a second check on selected locations of the patient during the second cycle (days 3 and 4). Of the five locations measured, the averaged dose was 201.9 cGy, which was 1.0% higher than the prescription dose and within our accepted tolerance (Table 3). Therefore the new *B*-factor and the calculated MUs were used for the remainder of the treatment course.

**Table 3:** OSL detector dose measurements for cycle two of first patient for reduced MUs

OSL Detector Location	AP + LPO + RPO Day 3 Dose (cGy)	PA + LAO + RAO Day 4 Dose (cGy)	Total Dose (cGy)	% Diff from Prescription
Forehead	96.1	117.2	213.3	6.6

Posterior of Head	121.9	84	205.9	3.0

Umbilicus	79.1	117.3	196.3	18

Posterior of Umbilicus	125.6	72.6	198.2	09

Back of Right Hand	70.5	125.3	195.7	21

**Average**			**201.9**	**1.0**

For the second pediatric TSEI patient, the adjusted *B*-factor determined from phantom and in-vivo measurements in the first patient was used for MU calculations. In-vivo dosimetry was performed as in the previous patient as part of patient-specific TSEI QA. The average dose determined by the OSL detectors was 198.3 cGy, 0.8% lower than the prescription. Based on these results, no adjustment was made to the dose calculations or patient setup. However, with this patient, individual regions including the top of the head and the inner thighs exceeded our ± 10% dose uniformity criteria. After discussing with the ain's physician, an electron boost was concurrently administered to the top of the patient's head, which had visible lesions, for the second half of the treatment course based on under dosage of this region.

## Discussion

In this work we present the commissioning and quality assurance processes used to successfully implement a pediatric TSEI program at UTSW. With this modified Stanford technique, the sedated pediatric patient is secured by a custom made harness and rotational system attached to a common TBI stand. Though pediatric leukemia cutis and the subsequent need for TSEI are rare, the ability to commission the technique on a modified TBI stand is appealing for clinical implementation. To the best of our knowledge this is the first complete description of the commissioning and QA of pediatric TSEI using a modified Stanford technique. Rubin et al. [12] use a similar pediatric TSEI technique for the treatment of acute monoblastic leukemia; however, it is presented as a medical case report with an incomplete description of the irradiation technique and the commissioning and QA methods. Pepek et al. [13] also present a medical case report for the treatment of leukemia cutis in pediatric patients. In this work they provide a brief description of the irradiation technique, which is based on a rotating platform at extended distances. The pediatric patients were of age where they were able to stand on their own in the required positions over the course of the treatment, thus removing the need for sedation and a rotating harness system. Earley et al. [7] describe, to date, the most complete description of the commissioning of a pediatric TSEI technique for the treatment of a sedated acute myelocytic leukemia patient. In this technique, the sedated patient is placed on a platform that is carried to the floor for AP and PA beams and the treatment couch for oblique beams (with the gantry moved to different angles). Beam profiles were acquired at a 200 cm distance for 6 MeV electrons to assess uniformity. From the authors' description, it seems the rotating harness system and irradiation technique described in our work would facilitate a faster total treatment time without having to move a patient platform in unison with an oxygen airway, IVs, and other lines for different beam positions.

Although commissioning showed overall surface dose uniformity within ± 10% in the plane perpendicular to the central axis for an adult patient's dimensions, there are many locations which could possibly exceed this uniformity criterion. Due primarily to surface irregularities, this has also been observed in patients treated by TSEI for mycosis fungoides. Surface cavities can receive less dose than flat or convex surfaces, while higher doses may occur in areas with body protrusions [21,22]. With the patient setup described in this work, in-vivo dosimetry demonstrated that under dose regions can occur when the skin is shielded by other body parts (inner thigh and leg regions), when the skin is shielded by patient vital sign monitoring equipment (beneath the blood pressure cuff), and at the top of the head. The inner thigh region underdose may be improved with the use of additional straps to separate the legs; however, a boost to the perineum region may still be needed at the discretion of the physician. In both UTSW clinical cases, every attempt was made to move oxygen tube and monitoring wires out of the treatment fields and away from the patient. The position of the blood pressure cuff was moved throughout the course of treatment to average out the shielding effect. As recommended in AAPM Report No. 23 [11], the use of boost fields required clinical judgment. Based on in-vivo dosimetry, the physician elected for a 6 MeV electron boost to the top of the head for the second patient with a 1 cm bolus since this region was under dosed during TSEI and this was the location of the isolated skin lesions.

When performing in-vivo dosimetry measurements to verify that the prescribed dose is delivered correctly and confirm dose uniformity, the choice of detector is important to consider. Thermoluminescent dosimeters have been the most commonly used for TSEI treatments due to the small volume minimizing the effect of dose gradients across the dosimeter. OSL dosimeters have recently been introduced for clinical dose measurements and have been implemented in clinical TSEI programs [23]. OSL dosimeters were used for in-vivo measurements due to their ease of use and quick readout with the MicroStar reader system.

Patient safety is of utmost important over the course of treatment and was of primary consideration in the commissioning process. It is crucial for the sedated patient in the harness system to remain in the same position while the treatment team is outside of the vault. During commissioning, filled water jugs were placed in the harness system to demonstrate structural integrity. Velcro straps as well as paper tape were on hand in abundance to set a consistent position. At every treatment, a team including an anesthesiologist, nurse, medical physicist, and radiation therapist were present to secure the patient, monitor the patient, verify treatment settings and setup, and deliver the required radiation dose. In addition to in-vivo dosimetry, machine QA procedures were implemented and performed on each treatment day to verify proper operation in TSEI mode. This consisted of checks for output, energy, symmetry, and flatness constancy. Due to the infrequent use of this treatment technique it is important, as part of the QA protocol, to verify any changes in the system over time which would affect the patient dose, including the absolute output calibration and *B*-factor. At our clinic, the second pediatric TSEI case for LC occurred approximately one year following the first case. Prior to the first treatment day of the second pediatric case, a calibrated parallel plate ionization chamber was used for absolute dosimetry in TSEI mode and the output for this mode was adjusted to the value used for initial commissioning.

## Competing interests

The authors declare that they have no competing interests.

## Authors' contributions

QB, JPD and FHH conducted the phantom measurement, initial commissioning of the setup, and treated the first patient. QB, BAH and FHH treated the second patient. QB and BAH drafted the manuscript. TDS oversaw the whole procedure. All authors read and approved the final manuscript.
